# High neuroticism is associated with common late adverse effects in a nationwide sample of long-term breast cancer survivors

**DOI:** 10.1007/s10549-023-07055-2

**Published:** 2023-08-01

**Authors:** Alv A. Dahl, Solveig K. Smedsland, Kathrine F. Vandraas, Synne K. Bøhn, Ragnhild S. Falk, Cecilie E. Kiserud, Kristin V. Reinertsen

**Affiliations:** 1 National Advisory Unit for Late Effects After Cancer Treatment, Department of Oncology, Oslo University Hospital, Radiumhospitalet, Nydalen, P.O. Box 4953, 0424 Oslo, Norway; 2 Oslo Centre for Biostatistics and Epidemiology, Research Support Services, Oslo University Hospital, Oslo, Norway

**Keywords:** Neuroticism, Breast cancer survivors, Late adverse effects, Health-related quality of life, Nation-wide cross-sectional study

## Abstract

**Purpose:**

Neuroticism is a basic personality trait characterized by negative emotions triggered by stress such as a breast cancer diagnosis and its treatment. Due to lack of relevant research, the purpose of this study was to examine if high neuroticism is associated with seven common late adverse effects (LAEs) in long-term (≥ 5 years) breast cancer survivors (BCSs).

**Methods:**

All female Norwegian BCSs aged 20–65 years when diagnosed with stage I–III breast cancer in 2011 or 2012 were invited to a questionnaire study in 2019 (*N* = 2803), of whom 48% participated (*N* = 1355). Neuroticism was self-rated using the abridged version of the Eysenck Personality Questionnaire, and scores dichotomized into high and low neuroticism. LAEs were defined by categorization of ratings on the EORTC QLQ-C30 (cognitive function, pain, and sleep problems) and QLQ-BR23 (arm problems) questionnaires, and categorizations of scale scores on mental distress, fatigue, and neuropathy. Associations between high neuroticism and LAEs were explored using multivariate logistic regression analyses.

**Results:**

High neuroticism was found in 40% (95%CI 37–42%) of BCSs. All LAEs were significantly more common among BCSs with high compared to low neuroticism. In multivariable analyses, high neuroticism was positively associated with all LAEs except neuropathy. Systemic treatment, somatic comorbidity, and not being in paid work were also significantly associated with all LAEs.

**Conclusions:**

High neuroticism is prevalent and associated with increased risks of LAEs among BCSs. Identification of high neuroticism could improve the follow-up care of BCSs as effective interventions for the condition exist.

**Supplementary Information:**

The online version contains supplementary material available at 10.1007/s10549-023-07055-2.

## Introduction

The population of long-term (≥ 5 years) breast cancer survivors (BCSs) is steadily increasing [1, 2]. BCSs face several challenges post-treatment due to their risk of cancer recurrence and late adverse effects (LAEs) that may have a negative impact on their current health status and overall quality of life [3–6]. Several factors associated with LAEs have been identified in BCSs such as young age at diagnosis, increasing treatment burden, comorbidity, mental distress, and socioeconomic factors [7].

Less research has explored the impact of basic personality traits on the prevalence of LAEs in BCSs. Among such personality traits, neuroticism is clearly the most important one concerning health and disease [8]. Neuroticism is defined as the propensity to experience negative emotions in reaction to stress, including anxiety, fear, anger, guilt, loneliness, worry, reduced self-esteem, and feelings of vulnerability [9]. Neuroticism is both hereditary and environmentally determined, and firmly established before young adult age. Thereafter, the individual level of neuroticism remains stable, but with some reduction in older age [10, 11]. Therefore, in the vulnerability-stress-reaction model, high neuroticism represents a vulnerability factor established early in life, affecting how individuals react and adapt to stressful life events, such as a breast cancer (BC) diagnosis. Recent studies have, however, shown that neuroticism is more modifiable than previously assumed, for example, by systematic interventions [12], and also by major stressors like being diagnosed and treated for cancer [13]. Studies of high neuroticism is, therefore, highly relevant for BCSs.

Associations between high neuroticism and increased occurrence of LAEs have been reported in survivors of testicular [14] and prostate cancer [15] but have not to our knowledge, been explored among long-term BCSs. The overall aim of the Norwegian nationwide cross-sectional SWEET study (**S**urvivorship-**W**ork-s**E**xual h**E**al**T**h-study) was to examine the impact of seven common LAEs (pain, arm problems, peripheral neuropathy, chronic fatigue, sleep problems, cognitive problems, and mental distress) on work life [16, 17] and sexual health [18] among long-term BCSs. Since the SWEET dataset also included self-ratings of neuroticism, we performed this sub-study with the following two aims: (1) To identify the prevalence of high neuroticism and explore differences in the prevalence of these LAEs between BCSs with high and low neuroticism; (2) To study the independent associations of neuroticism on LAEs. We hypothesized that BCSs with high neuroticism would have significantly more LAEs than those with low neuroticism, and that high neuroticism would be frequently associated with LAEs in multivariable analyses including other relevant explanatory variables.

## Materials and methods

### BCSs sampling

The design of the SWEET study has been described previously [16–18]. In short, the Cancer Registry of Norway (CRN) identified all Norwegian women diagnosed with BC stages I–III in 2011 or 2012 at the age of 20–65 years. To be included, women had to be free of pre- and post-malignancies, except for non-melanoma skin cancer and ductal carcinoma in situ. Among the 2803 BCSs identified and invited to participate, 1355 responded and were eligible (48%), of whom 1331 BCSs had complete scores on the neuroticism scale and were included in the analyses.

Information about non-responders (*N* = 1448) was limited to cancer-related data obtained from the CRN. Responders showed equivalent results as non-responders on all variables except for somewhat younger mean age at BC diagnosis, and higher rate of HER2 positivity and mean value for the proliferation marker Ki67 [18].

### Scales

*The abridged version of the Eysenck Personality Questionnaire* (EPQ-N) rated neuroticism with six items concerning long-term personality characteristics (Online Supplement) [19]. Each item on the EPQ-N was rated as present (1) or absent (0). The sum score ranged from 0 to 6, and higher sum score represented higher neuroticism. As the distribution of the sum scores was positively skewed, we used the established dichotomization of the sum score into high (sum score 3–6), and low (sum score 0–2) neuroticism groups [20]. Internal consistency expressed as Cronbach’s coefficient alpha was 0.76.

*The EORTC QLQ-C30* version 3 was used to assess aspects of generic health-related quality of life, and the items were rated on four points Likert scales from 1 (“not at all”) to 4 (“very much”). All scores were transformed to 0–100 scales according to the EORTC-scoring algorithms. Higher scores on the functional scales indicate better functioning, while higher scores on the symptom scales indicate higher symptom load [21]. Established separate cut-off values indicating clinically relevant problems with cognitive functioning, pain, and insomnia were used to define them as LAEs [22]. Cronbach’s alpha was ≥ 0.72 for these variables.

*The EORTC QLQ-BR23* was used to assess arm symptoms. The BC-specific module has the same scoring alternatives and algorithms as the QLQ-C30 [23]. Arm problems as a LAE were defined by a score ≥ 3 on any of the three items of pain, movement, or lymphedema.

*The Patient Health Questionnaire-9 (PHQ-9)* covered depression symptoms experienced during the last 2 weeks. Each item was scored from 0 (“not at all”) to 3 (“nearly every day”), providing a 0 to 27 severity sum score. A case of probable major depressive episode (MDE) was defined by a sum score ≥ 10 [24, 25]. Cronbach’s alpha was 0.85.

*The General Anxiety Disorder 7-item scale* (GAD-7) consists of seven items rating worry and anxiety symptoms during the last 2 weeks. Each item is scored from 0 (“not at all”) to 3 (“nearly every day”) with total scores ranging from 0 to 21. The cut-off score for a case of probable generalized anxiety disorder (GAD) was set at ≥ 10 [26]. Cronbach’s alpha was 0.86.

*Mental distress* as a LAE was defined as having probable MDE and/or GAD.

*Neuropathy* was assessed using two items from the Scale for Chemotherapy Induced Neurotoxicity (SCIN) [27]. The presence of peripheral sensory neuropathy in hands and feet, respectively, was rated from 0 (“not at all”) to 3 (“very much”), providing a sum score from 0 to 6. The sum score was dichotomized into high (≥ 4) and low (≤ 3) degree of neuropathy, where high degree was considered a LAE.

*The Fatigue Questionnaire* (FQ) includes 11 items measuring four mental and seven physical fatigue symptoms during the past month, scored from zero (“less than usual”) to three (“much more than usual”), with higher scores implying more fatigue. Scores on each item were dichotomized (0 = 0 or 1, 1 = 2 or 3), and cases with chronic fatigue identified by a sum score ≥ 4, and duration of complaints for 6 months or more [25, 28]. Cronbach’s alpha was 0.86 for mental and 0.92 for physical fatigue.

### Other variables

*Cancer-related variables* including age at diagnosis, BC characteristics and stage, and surgical treatment were retrieved from the Cancer Registry of Norway. Information on radiotherapy and on systemic treatments was self-reported and systemic treatments categorized as follows: no systemic treatment (“none,” reference), chemotherapy only, endocrine only, and chemo + endocrine therapy.

*Socio-demographic variables* included living with partner or not, basic education [long > 12 years (reference) versus short ≤ 12 years] and work status [“paid work” (reference) versus “not in paid work,” including those with disability pension or premature and age-defined retirement at 67 years]. *Somatic comorbid diseases* included self-reported cardiovascular, pulmonary, kidney, gastro-intestinal, rheumatic, or thyroid diseases, arthrosis, epilepsy, or diabetes. Number of comorbidities were categorized into none (reference), 1–2, and > 2 comorbid diseases. *Obesity* was defined as body mass index ≥ 30 kg/m^2^, calculated from self-reported height and body weight.

### Statistical methods

Continuous variables were described by mean and standard deviation (SD), and groups were compared using independent sample *t* tests. Categorical variables were given as numbers and percentages, and groups were compared with chi-square tests. Missing values were handled according to available guidelines or common practice for each of the scales.

Multivariable logistic regression analyses were used to investigate associations between neuroticism (high versus low) and the seven common LAEs. We performed adjustments for age at diagnosis, type of surgery (mastectomy versus breast conserving surgery), systemic treatments (none, chemotherapy only, endocrine only, chemo + endocrine), somatic comorbidity (none, 1–2, > 2), obesity, basic education (short versus long), and work status (paid work yes/no). No multicollinearity between the explanatory variables was observed. Interactions were explored by testing two-way interactions terms of the independent variables. The strength of associations was expressed as odds ratios (ORs) with 95% confidence intervals (95%CIs). Associations with *p* values < 0.05 were considered statistically significant, and all tests were two sided. All analyses were performed using IBM SPSS statistics version 28 (Armonk, NY).

## Results

### Characteristics of the BCSs

The mean age of BCSs at diagnosis was 52 years and mean time since diagnosis was 7.5 years, and 52% had long education, and 73% lived with a partner (Table 1).

**Table 1:** Cancer characteristics and somatic comorbidity among breast cancer survivors with high and low neuroticism and in the total sample at survey

Variables	High neuroticism (*N* = 526)	Low neuroticism (*N* = 805)	*p *value	Total sample (*N* = 1331)
Age at diagnosis (years)			–	
Mean (SD)	50.9 (9.0)	52.5 (8.3)	< **0.001**	51.9 (8.6)
Age at survey, mean (SD)	58.9 (9.1)	60.5 (8.3)	** < 0.001**	59.9 (8.7)
Years since diagnosis, mean (SD)	7.6 (0.6)	7.5 (0.7)	0.139	7.5 (0.6)
Stage, *N* (%)			0.430	
I	225 (43)	370 (46)		595 (45)
II	188 (36)	289 (36)		477 (36)
III	48 (9)	58 (7)		106 (8)
Missing	65 (12)	88 (11)		153 (11)
Surgery, *N* (%)			0.596	
Mastectomy	224 (43)	331 (41)		555 (42)
Breast-conserving therapy	302 (57)	474 (59)		776 (58)
Radiotherapy, *N* (%)	426 (81)	639 (79)	0.473	1065 (80)
Systemic treatments, *N* (%)			0.050	
None	82 (16)	155 (20)		237 (18)
Chemotherapy only	80 (15)	144 (18)		224 (17)
Endocrine only	64 (12)	106 (13)		170 (13)
Chemo + endocrine	297 (57)	391 (49)		688 (52)
Somatic comorbidity, *N* (%)			**0.004**	
None	148 (28)	280 (35)		428 (32)
1–2 diseases	285 (55)	429 (53)		714 (54)
≥ 3 diseases	90 (17)	95 (12)		185 (14)

Bold indicates *p* < 0.05

### Prevalence of high neuroticism and LAEs

Of 1331 BCS, 526 (40%, 95%CI 37–42%) had high neuroticism. The prevalence of LAEs ranged from 20 to 47%, of which mental distress was the least and pain the most frequent complaint (Table 2).

**Table 2:** Psychosocial characteristics and late adverse (LAEs) effect among breast cancer survivors with high and low neuroticism and in the total sample at survey

Variables	High neuroticism (*N* = 526)	Low neuroticism (*N* = 805)	*p* value	Total sample (*N* = 1331)
Living with partner, *N* (%)	382 (73)	595 (74)	0.603	977 (73)
Basic education, *N* (%)			**0.001**	
Short (≤ 12 years)	289 (56)	345 (43)		634 (48)
Long (> 12 years)	229 (44)	452 (57)		681 (52)
Income status, *N* (%)			** < 0.001**	
In paid work	175 (34)	371 (47)		546 (42)
Not in paid work	338 (66)	418 (53)		756 (58)
QLQ-C30-defined LAEs, *N* (%)				
Cognitive problems	323 (61)	255 (32)	** < 0.001**	578 (43)
Insomnia	277 (53)	173 (22)	** < 0.001**	450 (34)
Pain problems	336 (64)	288 (36)	** < 0.001**	624 (47)
QLQ-BR23-defined LAE, *N* (%)			** < 0.001**	
Arm problems	226 (43)	194 (24)		420 (32)
Chronic fatigue, *N* (%)	244 (47)	179 (23)	** < 0.001**	423 (32)
High degree neuropathy, *N* (%)	133 (26)	138 (18)	** < 0.001**	271 (21)
Generalized anxiety, *N* (%)	81 (16)	10 (1)	** < 0.001**	91 (7)
Major depression, *N* (%)	174 (34)	65 (8)	** < 0.001**	239 (18)
Mental distress, *N* (%)	194 (38)	67 (8)	** < 0.001**	261 (20)
Obesity (BMI ≥ 30 kg/m^2^), *N* (%)	120 (23)	116 (16)	** < 0.001**	236 (18)

Bold indicates *p* < 0.05

### Differences between BCSs with high and low neuroticism

BCSs with high neuroticism were younger at BC diagnosis, had more comorbid somatic diseases (Table 1), shorter education and a higher proportion were not in paid work (Table 2) compared to those with low neuroticism.

All LAEs were more frequent among BCSs with high neuroticism than among BCSs with low neuroticism. The proportion of the BCS with mental distress was 38% in those with high compared to 8% in those with low neuroticism. The prevalence of the other LAEs among BCSs with high neuroticism was 1.5–2.5-fold the prevalence among those with low neuroticism (Table 2).

### Multivariable analyses

After adjustments, neuroticism was associated with all the LAEs except neuropathy. BCSs with high neuroticism had 5.5-fold increased odds of mental distress compared to BCS with low neuroticism (OR 5.5, 95% CI 4.0–7.7). Further, BCSs with high neuroticism had a 3.5-fold risk for sleep problems, approximately a threefold risk for cognitive problems, pain, chronic fatigue, and a twofold risk for arm problems compared to those with low neuroticism. Systemic treatment with chemo- and endocrine therapy, somatic comorbidity, and not being in paid work were significantly associated with all LAEs, while younger age at survey was significantly associated with all LAEs except high neuropathy (Tables 3, 4). Mastectomy was significantly associated with pain and arm problems, and obesity with pain.

**Table 3:** Multivariable logistic regression analyses of independent variables and LAEs at survey as dependent variables

Variables	Cognitive problems			Pain problems			Insomnia			Mental distress		
	OR	95% CI	*p* value	OR	95% CI	*p* value	OR	95% CI	*p* value	OR	95% CI	*p* value
High neuroticism	2.98	2.32–3.85	** < 0.001**	2.72	2.10–3.53	** < 0.001**	3.54	2.73–4.59	** < 0.001**	5.53	3.96–7.71	** < 0.001**
Age at survey	0.94	0.92–0.96	** < 0.001**	0.97	0.95–0.99	** < 0.001**	0.96	0.94–0.98	** < 0.001**	0.93	0.91–0.95	** < 0.001**
Mastectomy	1.01	0.78–1.31	0.978	1.33	1.02–1.73	**0.035**	0.82	0.63–1.08	0.162	1.02	0.74–1.43	0.886
Systemic treatment			** < 0.001**			**0.040**			**0.006**			**0.048**
None (reference)	1.00	–	–	1.00	–	–	1.00	–	–	1.00	–	–
Chemotherapy only	1.34	0.87–2.07	0.187	1.54	0.98–2.41	0.060	0.89	0.56–1.43	0.635	1.94	1.04–3.64	**0.039**
Endocrine only	0.74	0.46–1.19	0.210	1.25	0.79–1.98	0.354	0.89	0.55–1.45	0.645	1.29	0.64–2.63	0.478
Chemo + endocrine	1.72	1.20–2.46	**0.003**	1.70	1.17–2.46	**0.005**	1.50	1.03–2.19	**0.035**	2.04	1.19–3.50	**0.010**
Somatic comorbidity			**0.020**			** < 0.001**			**0.002**			** < 0.001**
None (reference)	1.00	–	–	1.00	–	–	1.00	–	–	1.00	–	–
1–2 disease(s)	1.43	1.08–1.89	**0.013**	2.86	2.14–3.82	** < 0.001**	1.45	1.08–1.96	**0.015**	1.33	0.92–1.92	0.136
3 + diseases	1.65	1.08–2.50	**0.019**	7.38	4.71–11.56	** < 0.001**	2.08	1.36–3.19	** < 0.001**	2.78	1.66–4.66	** < 0.001**
Short education	1.01	0.78–1.30	0.964	1.21	0.93–1.56	0.156	1.08	0.83–1.41	0.569	0.94	0.67–1.30	0.696
Not in paid work	1.90	1.41–2.55	** < 0.001**	1.92	1.44–2.59	** < 0.001**	2.23	1.64–3.03	** < 0.001**	1.84	1.27–2.65	** < 0.001**
Obesity	1.08	0.78–1.48	0.641	1.89	1.36–2.64	** < 0.001**	0.84	0.60–1.17	0.296	0.95	0.64–1.41	0.796

Bold indicates *p* < 0.05

**Table 4:** Multivariable logistic regression analyses of independent variables and LAEs at survey as dependent variables

Variables	Arm problems			Chronic fatigue			High neuropathy		
	OR	95% CI	*p* value	OR	95% CI	*p* value	OR	95% CI	*p* value
High neuroticism	2.00	1.54–2.59	** < 0.001**	2.66	2.05–3.46	** < 0.001**	1.24	0.92–1.68	0.165
Age at survey	0.96	0.95–0.98	** < 0.001**	0.95	0.94–0.97	** < 0.001**	1.00	0.98–1.02	0.846
Mastectomy	1.48	1.13–1.92	**0.004**	0.89	0.68–1.17	0.413	1.23	0.91–1.67	0.180
Systemic treatment			**0.016**			**0.001**			** < 0.001**
None (reference)	1.00	–	–	1.00	–	–	1.00	–	–
Chemotherapy only	1.68	1.06–2.66	**0.026**	1.80	1.13–2.88	**0.014**	3.35	1.84–6.10	** < 0.001**
Endocrine only	0.91	0.54–1.51	0.709	0.93	0.55–1.56	0.771	0.94	0.45–1.93	0.856
Chemo + endocrine	1.57	1.07–2.34	**0.023**	1.89	1.27–2.80	**0.002**	4.26	2.50–7.26	< **0.001**
Somatic comorbidity			** < 0.001**			** < 0.001**			** < 0.001**
None (reference)	1.00	–	–	1.00	–	–	1.00	–	–
1–2 disease(s)	1.64	1.22–2.20	**0.001**	1.27	0.94–1.70	0.116	1.88	1.32–2.70	** < 0.001**
3 + diseases	2.85	1.87–4.35	** < 0.001**	2.51	1.64–3.86	** < 0.001**	3.01	1.85–4.90	** < 0.001**
Short education	1.03	0.79–1.34	0.843	0.97	0.74–1.26	0.809	1.36	1.00–1.84	0.050
Not in paid work	1.52	1.13–2.05	**0.006**	1.75	1.30–2.37	** < 0.001**	2.09	1.47–2.98	** < 0.001**
Obesity	1.18	0.85–1.62	0.327	1.04	0.75–1.46	0.798	1.16	0.80–1.68	0.429

Bold indicates *p* < 0.05

## Discussion

In this nation-wide sample of long-term BCSs, 40% had high neuroticism. All common LAEs were significantly more prevalent in BCSs with high compared to low neuroticism. BCS with high neuroticism were more than twofold more likely to have common LAEs than those with low neuroticism.

We consider the strong associations between high neuroticism and LAEs demonstrated in this study as new findings which could have considerable clinical relevance for both BC patients and survivors, and for health professionals working with this survivor population. In clinical practice, BCSs with high neuroticism may be seen as those requiring more time during consultations due to their needs for explanations, encouragement, and reassurance from health care professionals. These needs are unconscious to the survivors but may trigger negative attitudes from their health care professionals if they are not attentive to them. These professionals should also be aware that high neuroticism may be modified by a variety of psychotherapeutic and psychopharmacological interventions. Such interventions modify neuroticism more than spontaneous age-based changes [6, 8, 12, 13, 25, 29]. However, studies exploring such interventions for high neuroticism among BCSs have not been performed according to our knowledge.

In the general population, high neuroticism is a vulnerability factor that implies increased risk for unhealthy lifestyle [26, 30], and somatic diseases, particularly cardiovascular ones [27, 31], and mental disorders [9], dementia [28, 32], and suicide [29, 33]. High neuroticism may, thus, also considerably increase the illness burden in BCSs. Further, it has been proposed that if counseling by health care providers about a healthier lifestyle in BCSs shall be effective, high neuroticism must be considered [30, 34].

Our finding that 40% of the BCSs have high neuroticism may be compared to the significantly lower prevalence of 33% (95%CI 32–34%) of high neuroticism among Norwegian female norms found using the same cut-off score on the EPQ-N scale [16, 20]. This difference must, however, be considered in light of age differences between these samples as 87% of the BCSs and only 60% of the norms were ≥ 50 years at survey [31, 35]. Since the level neuroticism is somewhat reduced with age [5], we can tentatively conclude that the prevalence of high neuroticism is higher among BCSs than among norms. This observation warrants further investigation since we are unaware of other prevalence studies of high neuroticism in BCSs. It should be noted that other cut-off scores have also been applied leading to other prevalence rates of high neuroticism in other cancer populations [14, 15]. The clinical relevance of various prevalence rates seems obvious and should be checked in every study.

Associations between LAEs in cancer survivors and systemic treatments, somatic comorbidity, and age are all well established [32, 33, 36, 37]. We found that systemic treatment with both chemo- and endocrine therapy, somatic comorbidity, and not being in paid work were significantly associated with all the explored LAEs, while high neuroticism and younger age at survey were significantly associated with all LAEs except neuropathy.

The finding that all the explored LEAs were more common in BCSs with high versus low neuroticism is in accordance with results reported in survivors of testicular and prostate cancer [10, 11, 14, 15], and in long‑term survivors of childhood, adolescence, and young adult cancers among whom 28% had BC [34, 38]. The finding of an increased proportion of LAEs in survivors with high neuroticism should be investigated in survivors of other types of cancer for its eventual general conclusion.

The strong associations between not being in paid work and the occurrence of LAEs were not surprising to us. The association between not being in paid work and increasing age in this sample was low (data not shown). This finding, therefore, probably reflects our prior results that among BCSs in paid work at BC diagnosis, and still within working age, 27% were on disability pension eight years later, of whom 83% reported that their disability was due to the BC [12, 13].

A major strength of our study is the large population-based sample size and inclusion of a wide range of variables potentially associated with neuroticism. A further strength is the use of self-rating instruments with established psychometric properties. Although we could not match our BCSs with norms on age, we still consider these comparisons a strength of our study.

The 48% response rate of the study is in accordance with other response rates in current population-based studies [35, 39]. An obvious limitation is the unknown duration of the complaints we defined as LAEs, and another limitation is the lack of a trauma instrument in our questionnaire which would have provided us with the BCSs’ ratings of their cancer as a negative life event. The lack of such an instrument and the cross-sectional study design inhibited us from testing the hypothesis that being diagnosed with BC is a sufficient trauma to increase neuroticism since such testing requires measurements both pre- and post-BC diagnosis. Our findings must be interpreted with these limitations in mind.

In conclusion, high neuroticism is prevalent in BCSs, and significantly associated with the most common LAEs. Attention to neuroticism as a vulnerability factor for high prevalence of LAEs could, thus, be important to optimize the follow-up care of BCSs. Due to the high prevalence and negative consequences of high neuroticism, referral to interventions for BCSs with high neuroticism should be considered.

## Supplementary Information

Below is the link to the electronic supplementary material.

Supplementary file1 (DOCX 14 KB)

## Data Availability

The dataset generated and analyzed during the current study are not publicly available due to Norwegian data protection legislation but are available from the senior author KVR on reasonable request.
