# Supplementary material for: High neuroticism is associated with common late adverse effects in a nationwide sample of long-term breast cancer survivors
Source: Breast Cancer Res Treat. 2023 Aug 1;202(1):97–104. doi: 10.1007/s10549-023-07055-2 (PMC10504095; doi:10.1007/s10549-023-07055-2)
Supplement: Supplementary file 1 — Supplementary file1 (DOCX 14 KB) [file 10549_2023_7055_MOESM1_ESM.docx]

**Supplement.**

**The 6-item version of neuroticism based on the Eysenck Personality Questionnaire.**

**Instruction:** The items below concern how you usually behave, feel, or act. Please, set a ring round the number for either Yes or No for each item. Please, respond quickly and do not think too long about the meaning of each item.

| **Items** | **Yes** | **No** |
| --- | --- | --- |
| 1. Are you often worried? | 1 | 0 |
| 1. Are your feelings easily hurt? | 1 | 0 |
| 1. Do you often feel that you lose interest? | 1 | 0 |
| 1. Do you have nervous problems? | 1 | 0 |
| 1. Do you often feel tired and indifferent/unmotivated without reason? | 1 | 0 |
| 1. Do you worry that terrible things might happen? | 1 | 0 |

Low neuroticism: sum score 0 – 2; High neuroticism: sum score 3 – 6 (reference #16).
